# BRCA in Gastrointestinal Cancers: Current Treatments and Future Perspectives

**DOI:** 10.3390/cancers12113346

**Published:** 2020-11-12

**Authors:** Eleonora Molinaro, Kalliopi Andrikou, Andrea Casadei-Gardini, Giulia Rovesti

**Affiliations:** Department of Oncology and Hematology, Division of Oncology, University of Modena and Reggio Emilia, 41121 Modena, Italy; ele.molinaro.89@gmail.com (E.M.); k.andrikou@hotmail.com (K.A.); giulia.rovesti@gmail.com (G.R.)

**Keywords:** BRCA1, BRCA2, gastrointestinal cancers, HRD, pancreatic cancer, Olaparib, PARP inhibitors, surveillance

## Abstract

**Simple Summary:**

*BRCA* gene mutations are progressively gaining more attention in the context of gastrointestinal malignancies, especially in pancreatic cancer where their identification can have both therapeutic and surveillance relevance.

**Abstract:**

A strong association between pancreatic cancer and *BRCA1* and *BRCA2* mutations is documented. Based on promising results of breast and ovarian cancers, several clinical trials with poly (ADP-ribose) polymerase inhibitors (PARPi) are ongoing for gastrointestinal (GI) malignancies, especially for pancreatic cancer. Indeed, the POLO trial results provide promising and awaited changes for the pancreatic cancer therapeutic landscape. Contrariwise, for other gastrointestinal tumors, the rationale is currently only alleged. The role of *BRCA* mutation in gastrointestinal cancers is the subject of this review. In particular, we aim to provide the latest updates about novel therapeutic strategies that, exploiting DNA repair defects, promise to shape the future therapeutic scenario of GI cancers.

## 1. Introduction

**Figure 1:**
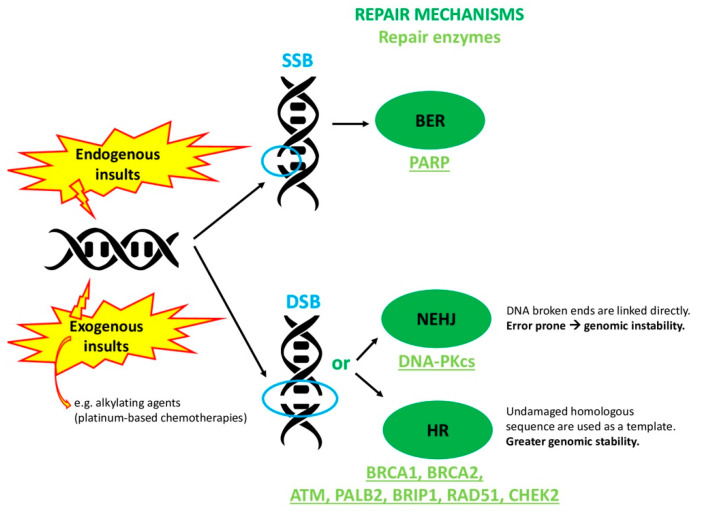
Types of DNA damage and repair mechanisms with related repair enzymes. A single-strand break (SSB) is accomplished by base excision repair (BER) through PARP enzymes. A double-strand break (DSB) is accomplished either with non-homologous end joining (NHEJ) through, mainly, DNA-dependent protein kinase (DNA-PKcs) or with homologous recombination (HR) through several enzymes (BRCA1, BRCA2, ATM, PALB2, BRIP1, RAD51, CHEK2).

*BRCA1* and *BRCA2* are famous tumor susceptibility genes. They encode for proteins playing a crucial role in the correct repair of damaged DNA. Indeed, these genes are key components of the homologous recombination (HR) pathway [1]. Particularly, during a normal cell cycle, the double-strand DNA can be damaged by internal and exogenous agents, producing a double-strand break (DSB). The most known mechanisms the cell uses to repair the DSB are the following: HR, as said, and non-homologous end joining (NHEJ). The first one allows a greater genomic stability compared to the second one. In fact, HR employs an undamaged homologous sequence as a template; instead, NHEJ links the DNA broken ends directly, without a template (Figure 1).

A pathogenic mutation in *BRCA1* or *BRCA2* genes causes an impaired HR. Therefore, the cell, being deficient in HR, utilizes NHEJ preferentially to repair the DSB. NHEJ, unlike HR, enhances the cellular genomic instability until carcinogenesis. For this reason, *BRCA* mutation carriers have a higher risk of developing cancers during their life [2].

**Figure 2:**
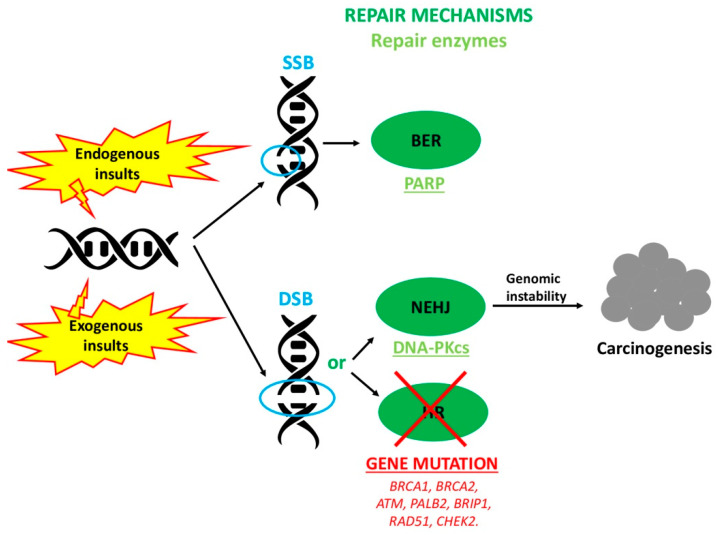
Effect of mutation in genes encoding for homologous recombination (HR) enzymes. A pathogenic mutation in *BRCA1, BRCA2, ATM, PALB2, BRIP1, RAD51* and *CHEK2* causes an impaired HR. The cell is, therefore, deficient in HR and it uses NHEJ preferentially to repair the DSB. However, NHEJ, unlike HR, enhances genomic instability until carcinogenesis.

In addition, several other proteins participate in the HR process such as PALB2, ATM, BRIP1, RAD51 and CHEK2 for the correct DSB repair. Particularly, ATM is a protein kinase able to find the DSB and monitor its reparation, PALB2 also has a modulatory role, stabilizing the BRCA2 protein, BRIP1 encodes a protein-terminal helicase 1 involved in the DSB repair machine, RAD51 forms a nucleoprotein filament catalyzing homologous pairing and CHEK2 blocks the cell cycle when a DSB occurs [3]. Consequently, also a mutation in these proteins determines a HR deficiency (HRD) and can produce the same effect as *BRCA1* and *BRCA2* pathogenic mutations (Figure 2).

All these proteins form the so-called BRCAness phenotype [4].

Overall, the diagnosis of a pathogenic mutation either in BRCA proteins or in BRCAness proteins is particularly important to establish a commensurate management of surveillance programs and treatment strategies in hereditary conditions [5,6].

**Figure 3:**
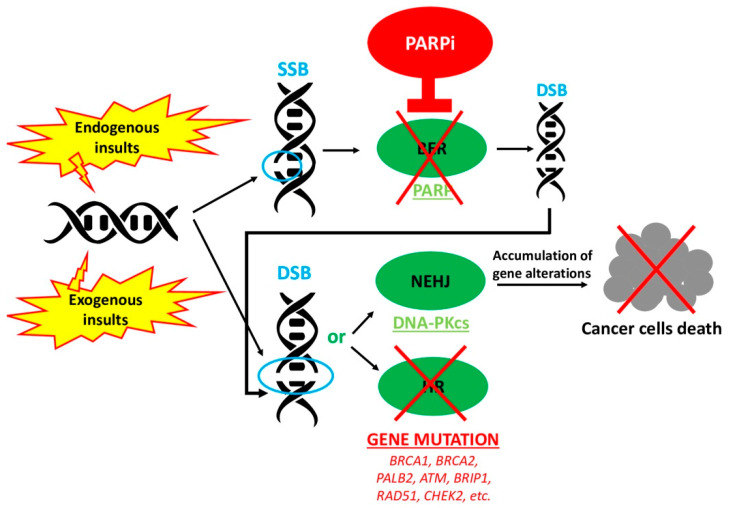
Mechanism of action of PARP inhibitors (PARPi). PARP inhibitors block PARP enzymes’ action, thus inducing an SSB to become a DSB. In the case of HR deficiency, a DSB cannot be repaired and therefore NHEJ is aberrantly activated, thus leading to accumulation of gene alterations until cancer cell death.

In recent years, due to the impairment of the HR pathway, *BRCA*-related cancers showed a major sensitivity to old and new drugs such as platinum-based chemotherapies and inhibitors of poly (ADP-ribose) polymerase (PARP), respectively [7]. As a matter of fact, platinum-based chemotherapies work as alkylating agents and produce a DSB in a cell unable to repair it [8]. Instead, PARP inhibitors (PARPi) are responsible for the so-called “synthetic lethality”. This process consists of different events that only when taken together can bring the cell to death. In detail, since also a single-strand DNA can be damaged, its reparation is mediated by PARP enzymes through the mechanism of base excision repair (BER). When this pathway is interrupted by the action of PARPi, the single-strand DNA break (SSB) cannot be repaired and it becomes a DSB. At last, in patients with a HR deficiency, such as *BRCA* mutation carriers, also a DSB cannot be repaired. Therefore, the cell accumulates gene alterations that lead it to death [9] (Figure 3).

Moreover, PARPi also act with an intrinsic cytotoxic effect, known as the “PARP trapping” effect, forming an inseparable complex with DNA strands [10].

As mentioned above, individuals carrying a germline mutation in *BRCA1* and *BRCA2* genes present a higher susceptibility to develop solid tumors. Classically, cancers most frequently associated with mutations in these genes are breast and ovarian ones. Indeed, the lifetime risk to develop breast cancer is approximately 52–72% among *BRCA1* mutation carriers and 45–84% among *BRCA2* mutation carriers. Furthermore, the lifetime risk for ovarian cancers is about 39–63% in *BRCA1* mutation carriers and 11–27% in *BRCA2* mutation carriers [11].

The cancer spectrum in *BRCA1* and *BRCA2* germline mutation carriers has been more extensively described in females than in males. In an effort to at least in part fill this gap, Silvestri et al. recently analyzed a large dataset of males harboring a germline mutation in the *BRCA1* or *BRCA2* gene, showing that being affected by any tumor and developing multiple cancers, particularly those of the breast, prostate and pancreas, is linked to a higher probability of being a *BRCA2*, rather than a *BRCA1*, carrier [12].

Regarding the topic of *BRCA* mutation, the knowledge about its role in gastrointestinal cancers is still limited, although it is known that the main underlining molecular pathways are those previously described; for this reason, the aim of this review is to describe the relationship existing between *BRCA* pathogenic variants and gastrointestinal cancers and its potential therapeutic role.

## 2. Pancreatic Cancer

With a five-year relative survival rate of 9%, the lowest among all cancer types, pancreatic cancer (PC) is the tumor with the most dismal prognosis. Moreover, deaths from PC are projected to increase dramatically in the next 20 years and by 2030, PC is expected to become the second leading cause of cancer-related death in the United States [13].

Cigarette smoking, increased body mass index, dietary factors, heavy alcohol consumption and a recent diagnosis of diabetes mellitus have been associated with increased pancreatic cancer risk [14,15,16,17], but inherited genetic factors also play an important role in pancreatic cancer risk [18]. It is estimated that 3% of PC cases derive from hereditary cancer syndromes (Peutz–Jeghers syndrome, PJS, ORPHA:2869, gene *LKB1/STK11*; hereditary pancreatitis, HP, ORPHA:676, gene *PRSS1*; familial atypical multiple mole melanoma, FAMMM, ORPHA:404,560, gene *CDKN2A*; hereditary breast and ovarian cancer syndrome, HBOCS, ORPHA:145, genes *BRCA1* and *BRCA2*; Lynch syndrome, LS, ORPHA:144, genes *MLH1*, *MSH2*, *MSH6*, *PMS2*; familial adenomatous polyposis, FAP, ORPHA:733, gene *APC*), and that another 4–10% of the cases are classified as familial PC (FPC), which is defined as an individual who has two or more first-degree relatives (FDRs) with PC and without association, with known hereditary genetic syndromes [19,20,21]. *BRCA* mutations are the most common germline genetic alterations known to occur in PC, inherited in an autosomal dominant pattern with incomplete penetrance [22]. *BRCA1* and *BRCA2* pathogenic mutations are found in 1% or less and in up to 2% of unselected PC cases, respectively [23,24,25,26]. Among Ashkenazi Jewish individuals with pancreatic cancer, these kinds of mutations are found in up to 13.7% of unselected cases. In FPC, *BRCA2* mutations are found in about 5% to 10% of cases and *BRCA1* mutations in approximately 1%. The lifetime risk of developing PC is 2.1–3.5 times higher in *BRCA* mutation carriers [27,28]. In particular, it is estimated to be 3% for carriers of mutations in *BRCA1* and 5% to 10% for carriers of mutations in *BRCA2* [23], certainly lower than the risk of developing breast or ovarian cancer [29].

As for breast and ovarian cancer, it is likely that mutations in a specific gene region may influence the risk and the characteristics of pancreatic cancer that is developed by *BRCA* mutation carriers; in their retrospective study of 5143 Italian families with history of *BRCA*-related malignancies, Toss A. et al. indicated two possible pancreatic cancer cluster regions (PCCR) that should be further verified in a larger cohort of *BRCA*-associated pancreatic cancer patients [11] and that are different from those previously identified for *BRCA1* in breast and ovarian cancers (BCCR and OCCR, respectively) and only marginally overlapping for *BRCA2*.

The prognosis of PC in *BRCA* mutation carriers remains unclear. In their retrospective analysis, Reiss et al. suggested a better prognosis in *BRCA1, BRCA2* or *PALB2* mutations carriers compared with non-carriers (21.8 vs. 8.1 months OS, HR 0.35, 95% CI 0.2–0.62; *p* < 0.001) [30]. However, some other studies showed no difference in overall survival (OS) [31,32] or even suggested a worse prognosis in *BRCA* mutation carriers [33]. Currently, systemic therapies for PC determine only a small increase in OS, therefore research advances are compelling, possibly moving in the direction of personalized, biomarker-driven options. Recent large-scale cancer genomic studies demonstrated a heterogeneous mutational profile, with activating mutations of *KRAS* present in over 90% of mutations of *TP53*, *CDKN2A* and *SMAD4* in over 50% of cases. Other mutations have been found with a prevalence of less than 5%, with frequent heterogeneity from case to case, thus involving a significant intertumoral heterogeneity. A whole-genome sequencing analysis of 100 PC patients showed that chromosomal structural variation is a relevant mechanism of DNA damage in pancreatic carcinogenesis, allowing the identification and classification of PC into four specific subtypes of pancreatic adenocarcinoma: stable, locally rearranged, scattered and unstable. The unstable subtype, exhibiting a large number (>200, maximum of 558) of structural variations, resulted in being associated with inactivation of homologous recombination DNA damage repair (HR-DDR) genes (*BRCA1*, *BRCA2* or *PALB2*) exhibiting a unique mutational signature reflecting defects in DNA maintenance and displaying sensitivity to DNA-damaging agents [34]. *KRAS*, *TP53*, *CDKN2A* and *SMAD4* are not currently actionable therapeutic targets, as the most commonly mutated genes. Notably, however, mutations in the DDR system, including *BRCA1* and *BRCA2*, but also *ATM* and *PALB2*, are emerging biologic targets for therapy in advanced pancreatic cancer [35].

### 2.1. BRCA Testing for Therapeutic Purpose

Identification of *BRCA1* and *BRCA2* mutations offers potential therapeutic advantages as they confer increased sensitivity to PARPi, reflecting a unique biology of *BRCA*-mutated pancreatic cancer cells [36,37].

The clinical evolution of PARPi in the context of PC has evolved from being used as monotherapies in refractory disease to maintenance therapies and in combination with other classes of therapeutics [36].

The only phase III trial has been conducted in the maintenance setting and is the international, randomized, placebo-controlled POLO (Pancreas cancer OLaparib Ongoing) trial, in which patients with metastatic pancreatic cancer and a germline *BRCA1* and/or *BRCA2* mutation whose disease had not progressed on first-line platinum-based chemotherapy derived a statistically significant and clinically meaningful improvement in progression-free survival (PFS; primary endpoint of the study) from maintenance treatment with PARP inhibitor olaparib vs. placebo. Median PFS was significantly longer in the olaparib arm (7.4 vs. 3.8 months, *p* = 0.004) and objective response rate (ORR) (23.1% vs. 11.5%) and median duration of response (24.9 vs. 3.7 months) were also improved. No difference in terms of median OS was observed between the two groups (18.9 vs. 18.1 months in the olaparib arm and placebo arm, respectively, *p* = 0.68), but data are still immature for this outcome since they derive from a planned interim analysis at data maturity of 46% [38]. Health-related quality of life (HRQoL) was preserved with maintenance olaparib treatment with no clinically meaningful difference compared with placebo, an important result for patients particularly when considering the cumulative toxicities of standard-of-care chemotherapies [39]. Of note, 21.7% of patients in the POLO trial progressed on first-line treatment and were ineligible for randomization, consistent with findings reported by Wattenberg et al. [40] in their retrospective real-world cohort study where over 40% of *BRCA*-defective PC patients did not respond to platinum-based chemotherapy and up to 20% had disease progression as best response even in the first-line setting. Clearly, a subset of PC patients harboring a *BRCA* germline mutation do not display the typical and possibly targetable HRD phenotype and the question on how to identify this subgroup of patients is still open [41]. Biomarker enrichment for the POLO trial was based on the presence of germline *BRCA1* and *BRCA2* mutations identified using the BRACAnalysis companion diagnostic assay; however, germline *BRCA1* and *BRCA2* mutations, which are typically found, respectively, in 1% and 2% of unselected PC cases, might reflect only the tip of the iceberg with regard to the potential target population [42]. Beyond germline *BRCA1* and *BRCA2* mutations, some *BRCA*-proficient tumors have defects in HR-DDR genes including *ATM*, *ATR*, *CHK1*, *CHK2*, *PALB2* and *RAD51* and also these cases, sharing the molecular features of *BRCA*-mutated tumors (BRCAness), are considered good targets for PARP inhibition treatment [21]. Nonetheless, on the basis of the randomized, placebo-controlled POLO trial, which showed that a biomarker-driven approach to PC treatment is achievable in practice, on December 27, 2019, the Food and Drug Administration (FDA) approved olaparib for the maintenance treatment of adult patients with deleterious or suspected deleterious germline *BRCA*-mutated metastatic pancreatic cancer, as detected by an FDA-approved test, whose disease has not progressed on at least 16 weeks of a first-line platinum-based chemotherapy regimen.

Outside the maintenance setting, the use of PARPi as single agents has generally underperformed in advanced-stage PC, suggesting that rational combination therapies are necessary in this disease and also relevant to the setting of PARP inhibitor resistance, which has both genomic mechanisms, such as *BRCA1* and *BRCA2* reversion mutations, and non-genomic mechanisms, including *ATR* pathway activation in order to bypass the impaired HR-DDR [35,43].

The combination of PARPi and chemotherapy has a strong rationale. Platinum-based drugs (cisplatin, carboplatin, oxaliplatin) are DNA cross-linking agents that kill tumor cells by interfering with DNA repair and inducing DSB, whereas topoisomerase inhibitors stall the replication fork by stabilizing the DNA complex in unrepaired state, enhancing SSBs. For this reason, the association between PARPi and these agents has been evaluated in several clinical trials. An open-label phase I/II clinical trial (NCT01489865) tested the combination of veliparib with mFOLFOX6 (modified Folinic acid + 5-FU + Oxaliplatin) in advanced PC patients. Patients in phase II of the trial were both pretreated (18 patients) and untreated (15 patients) and they were pre-selected for germline or somatic DDR mutations (69%) or had a family history suggestive of hereditary breast or ovarian cancer syndrome (HBOCS, 27%). The primary endpoint of the study was ORR, equal to 26% for the whole cohort. Interestingly, when considering different subgroups of patients separately, ORR was higher in platinum-naïve in respect to platinum-pretreated patients (33% vs. 7%, respectively), in patients with HBOCS in respect to no-HBOCS patients (30% vs. 14%, respectively) and in DDR mutation-positive in respect to negative ones (50% vs. 17%, respectively). The ORR for the platinum-naïve, HBOCS and DDR mutation-positive cohort was 58%, strongly highlighting the relevance of patient selection. Median PFS and OS for this selected cohort of patients was 8.7 and 11.8 months, respectively (vs. 3.7 and 8.5 months, respectively, for the unselected cohort of patients) [44]. The SWOG S1513 phase II trial (NCT02890355) randomized a biomarker unselected metastatic PC population to receive veliparib plus modified FOLFIRI (Folinic acid + 5-FU + Irinotecan) or FOLFIRI alone as second-line treatment. A total of 9% of patients had HRD genes mutations and 20% had other DDR genes, not classified as HRD, mutations. A planned interim futility analysis showed that the experimental arm did not have an OS benefit (5.1 vs. 5.9 months; HR 1.3, 95% CI 0.9–2.0, *p* = 0.21) for biomarker unselected patients, whereas it was likely to be superior to the control arm for patients with HRD in respect to patients without HRD. The incidence of grade 3/4 treatment-related adverse events (AEs), mainly fatigue, neutropenia and nausea, was higher in the veliparib plus chemotherapy arm [45]. The combination of mFOLFOX6 plus veliparib is promising, especially in highly selected patients who are platinum-naïve and have DDR mutation and HBOCS history, but the lack of direct comparison to chemotherapy alone limits the upfront use of this strategy. SWOG S1513 suggests that the inclusion of metastatic PC with any defect in the DNA maintenance system in clinical trials with irinotecan chemotherapy should be pursued. Both trials showed an increased toxicity profile with the combination arms, indicating that the benefit of the combination would potentially come at the cost of an increased toxicity. Further insights will probably be provided from the direct comparison of gemcitabine/cisplatin with and without veliparib in the front-line setting in *BRCA1/BRCA2*/*PALB2*-mutated PC [36].

Moving backwards to the neoadjuvant setting, Golan et al. recently showed that borderline resectable PC patients harboring a germline *BRCA* mutation have an increased chance of achieving a pathological complete response (44.4%, significantly higher than that reported for sporadic PC) and an improved survival after neoadjuvant treatment with FOLFIRINOX [46].

### 2.2. BRCA Testing for Surveillance Purposes

Based on the above-mentioned therapeutic implications of *BRCA1/BRCA2* mutation, the most recent update of the National Comprehensive Cancer Network (NCCN) Pancreatic Cancer guideline (v1. 2020) now recommends germline testing (on peripheral blood) for any patient in clinical practice with confirmed pancreatic cancer, using comprehensive gene panels for hereditary cancer syndromes and performing widely validated methodologies (next-generation sequencing—NGS). The response to PARPi and DNA-damaging agents in PC patients with somatic (tumor) mutation in one or more DNA damage response genes has been evaluated by Lowery et al., who concluded that the presence of those genes failed to improve patient’s response to platinum-based chemotherapies [47]. The mosaicism and heterogeneity of tumor HRD that might be present in the setting of somatic mutations might represent one of the reasons for that result. Clearly, further studies are needed to understand the degree of HRD that somatic mutations might confer since, reasonably, somatic mutations within the HRD pathway and also the BRCAness phenotype are likely to further expand the proportion of PC patients that might benefit from HRD-directed therapies [48].

A genetic testing proposal should occur providing a deep and comprehensive knowledge on all aspects related to possible test results and respecting the decisional time of the patient. Genetic counseling is then recommended for patients who test positive for a pathogenic mutation or for patients with a positive family history of cancer, especially pancreatic cancer, regardless of mutation status [49]. According to the American Society of Clinical Oncology (ASCO), genetic testing is recommended for both affected and unaffected individuals from familial PC families and families with at least three cases of PC diagnoses, but also for individuals for whom testing criteria for hereditary cancer syndromes with high risk of PC are met. Of note, ASCO also suggests that genetic testing should be discussed with any individual diagnosed with pancreatic cancer, even in the presence of an unremarkable family history [50]. Thus, there is now movement in clinical practice toward genetic testing for all patients with pancreatic adenocarcinoma.

Clinical management for PC probands necessarily raises the problem of surveillance, including counseling, for unaffected relatives. The main purpose of surveillance for high-risk individuals (HRIs) is the detection of precursor lesions or early PC, which is the only point at which a surgical (curative) approach may be feasible at present. However, standard screening procedures have not been settled and it is not clear whether screening offers clinical benefit. Numerous studies in the past have failed to show a substantial benefit of screening for pancreatic cancer [21]. More recent studies offered suggestions of benefit in at least some high-risk patients and it is reasonable that stronger evidence supporting surveillance in HRIs derive from long-term follow-up studies compared to single-round ones [51,52,53,54,55]. Currently, no protocols are established, and disagreement remains as to the best screening modality, time of screening initiation or follow-up duration. Nonetheless, there is general agreement that screening is appropriate for individuals at highest risk of developing pancreatic cancer [23]. Some of the promising and conceivable criteria for screening are based upon the International Cancer of the Pancreas Screening (CAPS) Consortium consensus (Table 1) [56,57].

High-risk patients should perform endoscopic ultrasound (EUS) and magnetic resonance imaging (MRI)/magnetic resonance cholangiopancreatography (MRCP), but the scientific community has not reached a consensus on the optimal ages to begin screening or the appropriate screening interval. Generally, it is recommended to begin surveillance at age 50 (or 10 years earlier than the age of the youngest affected relative), with a level of evidence of IV (based on a retrospective cohort or case–control studies) [57]. According to a systematic review including five prospective controlled studies for familial high-risk individuals, subjects in a screening program, mainly by EUS, had a significantly higher curative resection rate (60% vs. 25%) and longer median OS (14.5 months vs. 4.0 months) compared with the control group, although economic and emotional impacts were adverse in the screening group [58]. Regarding the psychological burden of surveillance in particular, in their multicenter prospective trial with follow-up data up to three years, Konings et al. found instead that high-risk individuals feared their next investigation less with the progression of surveillance, with decreasing worries about possible cancer diagnosis and normal or stable levels of depression and anxiety [59]. Accordingly, Paiella et al. stated that PC annual screening with MRCP seemed not to negatively influence HRIs’ psychological wellbeing, with the exception of younger subjects showing higher level of stress [60].

Even in the case a suspicious lesion is detected, no consensus has been reached with respect to the extension of pancreatic resection (partial or total pancreatectomy). In this setting, a multidisciplinary team is needed and surgical intervention must be individualized. In gene mutation carriers without any precursor lesion, prophylactic pancreatectomy is not indicated [57].

Beyond directing pancreatic screening and treatment decision, there are other recognized benefits for genetic testing. Foremost is that the identification of a mutation in the patient will allow for cascade testing of at-risk family members for the same mutation with limited cost and high accuracy. Family members without the mutation will not need pancreatic cancer screening, whereas those with the mutation may. Secondly, identification of a responsible mutation also provides information on other possible cancer risks associated with the mutation/syndrome. For each of the clinically actionable genes on these testing panels, guidelines to direct screening of at-risk individuals (e.g., for breast, ovarian and prostate cancers in *BRCA* mutation carriers or colon, endometrial and other cancers in Lynch syndrome mutation carriers) are available. In some cases, prophylactic surgery or chemoprevention may also be offered [23].

## 3. Other Gastrointestinal Cancers

The association between pathogenic mutations in *BRCA1* and *BRCA2* and other gastrointestinal tumors such as colorectal, gastric cancers, cholangiocarcinoma and hepatocellular carcinoma is unclear. Several population studies conducted over several years reported contradictory results. Therefore, these tumors are not accounted as criteria to select patients for *BRCA* genetic testing.

### 3.1. Colorectal Cancer

Colorectal cancer (CRC) is the second cause of cancer death in both men and women in the world. In most cases, CRCs are sporadic. However, different hereditary CRC syndromes exist; the best known are LS (or hereditary nonpolyposis CRC) and FAP. The first one is associated with a mutation in mismatch repair (MMR) genes, or rather *MLH1*, *MSH2*, *MSH6* or *PMS2* [61]. Lynch syndrome-related CRCs represent about 1%–3% of all CRC cases. FAP syndrome is caused by a germline mutation in the *APC* (adenomatous polyposis coli) gene and it is responsible for nearly 1% of all CRCs [62]. The link between CRC and a mutation in *BRCA1/BRCA2* genes is less coded; in fact, individuals affected by CRC are not normally tested either for *BRCA1* or for *BRCA2*.

In 1994, the Breast Cancer Linkage Consortium (BCLC) highlighted a statistically significant increased risk of CRC in a population of *BRCA1* mutation carriers (RR = 4.11, 95% CI 2.36–7.15) [63]. The same result was not observed in *BRCA2* mutation carriers. In subsequent years, different groups of investigators confirmed or disproved these findings. Thompson and Easton showed a 2-fold increased risk of colon cancer (RR = 2.03, 95% CI 1.45–2.85) and a decreased risk of rectal cancer (RR = 0.23, 95% CI 0.09–0.59) in *BRCA1* mutation carriers [64]. Moreover, Brose et al. underlined a 2-fold increased risk (11%, 95% CI 8.2%–13.2%) to develop CRC in *BRCA1* carriers compared to the risk reported by Surveillance, Epidemiology, and End Results (SEER) [65]. Phelan and his collaborators screened 7015 women carrying a *BRCA* mutation and found twenty-one CRC cases, an incidence not higher than that of the general population. Nevertheless, they observed an increased risk to develop CRC in women younger than 50 years carrying a *BRCA1* pathogenic mutation. Instead, no differences with global population rates were reported in older women and in *BRCA2* mutation carriers [66]. These findings are consistent with the results of other studies. Indeed, in a Polish population of 2398 unselected patients affected by CRC, Suchy et al. reported a mutation detection rate of about 0.42% (not higher than the rate of the control group, that was 0.48%). However, also in this trial, a major incidence of CRC in patients younger than 60 years (OR = 1.7) was underlined, though not statistically significant (*p* = 0.3) [67]. Thus, women with a *BRCA1* mutation should undergo a CRC screening test, such as a high-sensitivity fecal occult blood test or colonoscopy at a younger age. Notably, they may also be good candidates for chemoprevention programs with low-dose aspirin. Indeed, several trials highlighted a decrease in CRC incidence in subjects taking daily aspirin at the dosage of ≥75 mg/day [68]. Particularly, Burn et al. tested 600 mg/day of aspirin in patients with Lynch syndrome and observed a reduction in CRC incidence [69]. However, since the regular use of acetylsalicylic acid might cause severe AEs such as cerebral and gastrointestinal bleedings, the US Preventive Services Task Force recommends chemoprevention only for high-risk individuals, e.g., Lynch syndrome or FAP individuals [70].

Mersch et al. investigated the risk of developing CRC in a group of 613 *BRCA1* and 459 *BRCA2* mutation carriers and found no statistically significant difference between carriers and non-carriers [71]. Moreover, in a cohort study, Lin and colleagues observed no significantly different risk in 164 *BRCA1* and 88 *BRCA2* mutation carriers compared to the general population [72]. Other studies investigated if a family history of breast cancer was associated with a higher CRC incidence. Niell and colleagues did not identify a correlation between a family history of breast cancer in a first-degree female relative and the risk of developing CRC [73]. Conversely, Slattery and Kerber reported a low, but statistically significant, increased risk for CRC in patients with a positive family history for breast cancer [74]. Of note, several hereditary syndromes could be involved and could explain the association. Peutz–Jeghers syndrome, Cowden syndrome and Muir–Torre syndrome are just some examples of inherited conditions with a spectrum of diseases in which both cancers (breast cancer and CRC) are accounted. Furthermore, *APC* polymorphism I1307K, as reported by Woodage et al. and Redston and colleagues, might be associated with low penetrance to breast cancer susceptibility [75,76].

In summary, some family-based studies and prospective cohort studies suggested a possible greater risk among early-onset CRCs in *BRCA1* mutation carriers. These results seem to be confirmed by a systematic review and meta-analysis underlining a 1.49-fold higher risk of CRC in *BRCA1* mutation carriers [77]. Nevertheless, more studies are needed to investigate the real linkage.

In recent years, also in the context of CRC, new and old drugs demonstrated efficacy in HRD conditions. In their case report, Lin et al. described a complete pathological response in a young man affected by rectal cancer carrying a *BRCA2* pathogenic mutation with a platinum-based neoadjuvant chemotherapy [78]. The authors also reported a high tumor mutational burden (TMB), investigated by next-generation sequencing (NGS), without microsatellite instability (MSI), in their patient. Therefore, based on previous evidence for other cancers, they speculated also a possible rationale for the use of checkpoint inhibitors [78,79,80]. In their study involving 6396 CRC tumor samples, Naseem et al. recently detected *BRCA1* and *BRCA2* mutations in 1.1% and 2.8% of tumors, respectively. Interestingly, they found a higher frequency of *BRCA1* and *BRCA2* mutations in MSI-high (MSI-H) patients and found that those mutations were independently associated with higher TMB. Therefore, Naseem et al. also came to the conclusion that *BRCA1* and *BRCA2* mutations might potentially be predictive biomarkers for checkpoint inhibitors in CRC [81]. Notably, Harpaz and collaborators found a statistically significantly higher incidence of *BRCA* mutations and a higher TMB in CRCs with mucinous histology, compared to adenocarcinomas, suggesting that this association might lead to the use of histopathologic characterization, besides other tests, to identify patients who may be good candidates for immunotherapy [82].

As previously mentioned, PARPi are novel therapeutic agents. They currently play a very important role mostly in the treatment of ovarian cancer. Earlier studies investigated the role of ABT-888 (veliparib) in a CRC cell line pretreated with DNA-damaging chemotherapy agents, such as irinotecan and oxaliplatin [83], reporting a synergistic effect. ABT-888 showed a synergistic effect also in combination with radiation [84]. Based on these results, a phase II open-label study evaluating the action of veliparib in combination with temozolomide in metastatic CRC patients successfully met its primary endpoint with a disease control rate (DCR) of 24% and two confirmed partial responses [85]. However, PARPi demonstrated their efficacy also when a mutation occurred in the so-called BRCAness genes, such as in *ATM*. Wang et al. highlighted that CRC cell lines with an *ATM*-inactivating mutation had an increased sensitivity to olaparib [86].

Based on previous studies on myeloid malignancy [87], Leichman et al. tested a PARPi in microsatellite-stable (MSS) and -unstable (MSI) CRC patients in a phase II clinical trial. No differences between the two groups were observed. Therefore, the authors reported that microsatellite status is not a predictive marker of response to PARPi [88]. Certainly, more studies are needed, and for the time being, PARPi are not approved for the treatment of CRC.

### 3.2. Gastric Cancer

Gastric cancer (GC) is a heterogeneous disease, mostly sporadic, but hereditary in a small percentage of cases (1%–3%). Familial intestinal GC (FIGC) and hereditary diffuse GC (HDGC, ORPHA: 26106) are the principal hereditary GC conditions. HDGC syndrome is caused by a mutation in *CDH1*, the gene encoding for the E-cadherin protein, and it is characterized by an association between signet ring cell/diffuse GC and lobular breast cancer [89]. GC is also a key component of other hereditary cancer syndromes such as Lynch Syndrome, Li–Fraumeni syndrome (ORPHA:524, gene *TP53*) and Peutz–Jeghers Syndrome. Furthermore, GC is accounted in hereditary breast/ovarian cancer syndrome (HBOCS).

The BCLC reported a 6-fold increased risk of GC among first-degree relatives of both *BRCA* genes mutation carriers [28,63]. Brose et al. estimated a 4-fold higher lifetime risk to develop GC in *BRCA1* mutation carriers [65]. Tulinius and colleagues investigated the risk of developing GC in 995 women and found a 2-fold greater risk in the *BRCA2* mutation-positive cohort [90]. Conversely, van Asperen and collaborators highlighted no statistically significant higher risk of developing GC in *BRCA2* families in the Dutch population [91]. Some authors explained the contradictory results, arguing that breast and ovarian cancers have an earlier onset in *BRCA1/BRCA2* mutation carriers, therefore patients might not have time to develop GC afterwards. Notably, in their population-based study, Bermejo and colleagues found a major incidence of GC in males. Particularly, in 23 families with ovarian, breast and gastric cancers, they reported 23 GC cases in males and only 1 case in females [92]. Previously, also BCLC suggested a sex-related increased incidence of GC in males [28].

As mentioned above, an impairment in the proteins involved in HR causes a higher susceptibility to PARPi. A phase II study reported a significant improvement in OS with the combination of olaparib plus paclitaxel in Asian patients with advanced GC, especially in *ATM* mutation carriers [93]. A subsequent phase III trial (GOLD trial) did not confirm these results, showing an OS of 8.8 months (95% CI 7.4–9.6) in the olaparib group vs. 6.9 months (95% CI 6.3–7.9) in the placebo group. Moreover, among the *ATM* mutation carriers, the OS was 12 months (95% CI 7.8–18.1) in the experimental arm vs. 10 months (95% CI 6.4–13.3) in the standard arm [94].

Several other combination therapies were investigated. A phase II basket study demonstrated the tolerability and the reasonable efficacy (ORR 10%) of the association between olaparib and durvalumab, an anti PD-L1 monoclonal antibody, in patients with relapsed GC [95]. Currently, a study evaluating the combination of olaparib and ramucirumab (an angiogenesis inhibitor) is ongoing [96].

### 3.3. Cholangiocarcinoma and Hepatocellular Carcinoma

Cholangiocarcinoma (CCA) is the second most common hepatic neoplasm after hepatocellular carcinoma. In recent years, with the purpose of improving CCA treatment, Nakamura et al. found in a series of CCA several somatic alterations in potentially targetable genes, such as kinases *FGFR1*, *FGFR2*, *FGFR3*, *AKT3*, *BRAF*, *PIK3CA*, *EGFR* and *ALK* and oncogenes *MDM2*, *CCND3*, *CCND1*, *IDH1* and *IDH2*, but also in the tumor suppressor proteins BRCA1 and BRCA2 [97]. Moreover, Churi and his collaborators also highlighted targetable somatic mutations in *MSH2*, *MLH1*, *ATM*, *BAP1*, *MSH6*, *BRCA1* and *BRCA2* in 74 CCA cases [98]. The role of *BRCA1* and *BRCA2* in the pathogenesis of CCA was primarily suggested by BCLC. In 1999, they described a relative risk (RR) of developing CCA of about 4.97 (95% CI 1.50–16.52) among *BRCA2* mutation carriers [28]. Encouraged by these results, Golan and other authors identified 18 cases of CCA with genetic alterations in *BRCA1* and *BRCA2* genes: five of those were germline, thirteen were somatic mutations. Thirteen CCA patients were treated with a platinum-based chemotherapy and four patients received PARPi. Notably, one of the patients treated with PARPi experienced a progression-free survival of 42.6 months [99]. Cheng et al. also described a good response with olaparib monotherapy in a patient affected by intrahepatic CCA [100]. Recently, Spizzo et al. analyzed 1288 CCA samples and detected *BRCA1* and *BRCA2* mutations in 46 cases, at 3.6% (0.6% *BRCA1* and 3% *BRCA2*). They also underscored that these mutations were associated with a high mutational burden and suggested a potential rationale for the combination of PARPi with immunotherapies [101]. More evidence for the use of PARPi in CCA comes from pre-clinical experiences. Indeed, Fehling et al. highlighted a synergistic action of BET inhibitors (JQ1) with PARPi in CCA cell lines [102]. Another pre-clinical study suggested a potential role of olaparib in sensitizing CCA cells to radiation [103], as reported above for CRC. Moving from the benchside to the bedside, clinical trials are currently ongoing. For instance, a phase II trial is evaluating olaparib in patients with metastatic CCA and aberrant DNA repair genes (*BRCA1*, *BRCA2*, *ATM*, *RAD51* and others) [104]. The results of this trial, which are expected in 2021, and those from other trials are needed to clarify, firstly, the real role of *BRCA1* and *BRCA2* mutations in CCA, and secondly, the potential role for PARPi use either in monotherapy or in combination with other drugs.

Regarding hepatocellular carcinoma (HCC), evidence about its link with *BRCA1* and *BRCA2* mutations is extremely limited. In a recent study, Lin J. and colleagues analyzed a population of 357 patients with primary liver cancers: 214 HCC, 122 CCA and 21 mixed HCC and CCA. They found a mutation in *BRCA1* or *BRCA2* genes only in five HCC patients. However, they reported an ATM mutation rate of 6.07%, higher than that of CCA. Clearly, because of the scarce data available, the real role of BRCA proteins in the pathogenesis of HCC cannot be postulated [105].

### 3.4. Gastrointestinal Cancer Minorities

Lastly, but not least, coming to gastrointestinal cancer minorities, evidence is still little or absent. Recently, Hännimen et al. [106] and Quaas et al. [107] highlighted a possible role of *BRCA1* and *BRCA2* mutations in small bowel cancer pathogenesis, but data are very preliminary. The linkage between gastrointestinal tumors and *BRCA* mutation is currently drawing more and more attention and since PARPi might open a new therapeutic scenario in a wide range of cancers, potentially also in rare and orphan ones, evidence is expected to increasingly grow in the near future.

## 4. Future Perspectives

Remarkable progress on the genomic profiling of gastrointestinal cancers has been achieved in recent years. Based on encouraging results in breast and ovarian cancers, understanding the role of *BRCA1/BRCA2* mutations in the pathogenesis, prognosis and therapeutic decision of gastrointestinal malignancies, especially in PC, has gained particular interest. Moreover, the presence of *BRCA1/BRCA2* mutations is associated with increased risk for pancreatic cancer [108,109], but its role in the other gastrointestinal malignancies is still to be defined. In addition, the effect of *BRAC1/2* mutations on the survival of these patients remains controversial in the literature [109,110]. Therefore, large-scale genetic testing seems to be necessary in order to clarify doubts and broaden our knowledge in this field.

As mentioned above, increased evidence underlines the potential role of *BRCA1/BRCA2* mutations as predictive markers for the efficacy of platinum-based agents and other treatments in various types of cancer because of DNA repair defects [34,37,111,112]. In particular, pathological complete responses to platinum agents have been described in patients affected by *BRCA*-defective gastrointestinal tumors, whereas their efficacy in gastrointestinal tumors with somatic *BRCA* mutations still remains controversial [113,114]. Actually, several clinical trials are ongoing to evaluate the association between *BRCA1*/*BRCA2* gene mutations and platinum sensitivity in several tumors including gastrointestinal malignancies (Table 2).

Based on promising results of breast and ovarian cancers [115,116], several clinical trials of PARPi are undergoing for gastrointestinal malignancies, especially for pancreatic cancers. Recently, a phase III trial demonstrated that PARPi can significantly improve outcomes as maintenance treatment in patients affected by platinum-sensitive advanced pancreatic cancer harboring germline *BRCA* mutations [38]. However, the effect of these novel agents in tumors with somatic *BRCA* mutations is still not well defined. Therefore, randomized trials of PARPi alone or in association with chemotherapy in gastrointestinal tumors are ongoing; their results are expected to clarify the position of PARPi in the therapeutic armamentarium of these tumors.

Considering the relationship between high mutational burden and *BRCA* mutations reported for breast and ovarian cancers, these characteristics provide a rationale for the evaluation of immune checkpoint inhibitors in *BRCA*-mutated tumors [117,118]. However, further studies are required to better define the potential clinical benefit of immunotherapy alone or in combination with PARPi or platinum agents on this subset of gastrointestinal tumors. Hopefully, several ongoing trials will answer some of these unresolved issues, trying to improve the therapeutic landscape of GI malignancies in the coming years (Table 2).

Challenges for the future are definitely many. Firstly, the genetic background of patients affected by gastrointestinal tumors with *BRCA* mutations seems to be heterogeneous and underexamined and many questions remain to be explored; use of sophisticated software risk assessment tools may facilitate their better identification. Secondly, additional studies are necessary to better clarify the prevalence and penetrance of *BRCA1/BRCA2* mutations, and whether they might not be driver but rather passenger mutations only, and consequently to define the impact of available genomic information with clinical outcomes and eventually of personalized therapeutic approaches. Thirdly, with the advent of emerging agents (PARPi and immunotherapy) and their possible association with cytotoxic agents, further studies are necessary to define the most suitable use of such agents depending on disease status. Fourthly, since only a subset of patients with *BRCA* mutations seems to benefit from available treatments, there is a compelling need to identify predictive biomarkers able to direct the right treatment to the right patient. Finally, since clinical and genomic data about *BRCA*-mutated gastrointestinal cancers are still limited, though progressively expanding, international spreading of hereditary gastrointestinal tumors registries seems to be crucial for future studies.

## 5. Conclusions

In summary, a strong association between pancreatic cancer and *BRCA1* and *BRCA2* mutations is documented. Indeed, the POLO trial results can change the approach to newly diagnosed pancreatic cancers. Contrariwise, for other gastrointestinal tumors, the association is currently only alleged. Some colorectal epidemiological studies speculate a greater incidence of colorectal cancer in women younger than 50 years carrying a *BRCA1* mutation. A lower link emerged for gastric cancer and cholangiocarcinoma. Notably, for gastric cancer, a major incidence in males is presumed. Several pre-clinical studies and clinical trials, also in the absence of a genetic predisposition, investigated the effects of PARPi in combination with DNA-harmful agents (e.g., radiation, chemotherapies), which appear to be amplified in *BRCA*-defective patients.

## Figures and Tables

**Table 1:** Surveillance criteria according to the International Cancer of the Pancreas Screening (CAPS) Consortium consensus [56].

CAPS Consortium Consensus for Surveillance of HRIs.
Individuals with at least three or more affected relatives, of whom at least one is an FDR to the individual considered for screening
Individuals with at least two affected relatives who are FDRs to each other, of whom at least one is an FDR to the individual considered for surveillance
Individuals with at least two affected relatives on the same side of the family, of whom at least one is an FDR to the individual considered for surveillance
*LKB1/STK11* mutation carriers (PJS) regardless of family history
*CDKN2A* mutation carriers regardless of family history
*BRCA1* mutation carriers with one affected FDR
*BRCA2* mutation carriers with one affected FDR (or two affected family members, no FDR) with PC
*PALB2* mutation carriers with one affected FDR
MMR gene mutation carriers (LS) with one affected FDR
*ATM* mutation carriers with one affected FDR

**Table 2:** Summary of ongoing clinical trials in *BRCA*-mutated gastrointestinal tumors.

NCI Trial Number	Intervention	Cancer	Primary Endpoint	Phase
NCT03337087	Nal-IRI+ Fluorouracil + Rucaparib	Pancreatic, colorectal, gastroesophageal or biliary cancer	Toxicity, ORR, best response rate	I, II
NCT03838406	FOLFOX/CAPOX	Gastric cancer	ORR	Not Applicable
NCT03565991	Talazoparib + Avelumab	Advanced solid tumors	OR	II
NCT02286687	Talazoparib	Recurrent, refractory, advanced or metastatic cancers	Clinical benefit	II
NCT01989546	BMN 673 (Talazoparib)	Advanced solid tumors	Pharmacodynamic effect of talazoparib; response rate	I, II
NCT03140670	Rucaparib	Pancreatic cancer	Safety	II
NCT03428802	Pembrolizumab	Advanced solid tumors	Response rate	II
NCT02723864	Veliparib + VX-970 + Cisplatin	Refractory solid tumors	Safety, MTD, tolerability	I
NCT04182516	NMS-03305293	Advanced/metastatic solid tumors	Toxicity	I
NCT03875313	CB-839 + Talazoparib	Solid tumors	Safety, MTD, tolerability	I, II

ORR: objective response rate; OR: objective response; MTD: maximum tolerated dose.
